# Effect of the smartphone application on caesarean section in women with overweight and obesity: a randomized controlled trial in China

**DOI:** 10.1186/s12884-023-06004-7

**Published:** 2023-10-23

**Authors:** Yi Feng, Cuixia Shi, Chengyan Zhang, Chenghong Yin, Li Zhou

**Affiliations:** Department of Obstetrics, Beijing Obstetrics and Gynecology Hospital, Capital Medical University, Beijing Maternal and Child Health Care Hospital, No.251 Yaojiayuan Road, Beijing, 100026 China

**Keywords:** Smartphone, Obesity, Overweight, Pregnancy, Cesarean section, Gestational weight gain

## Abstract

**Background:**

The rate of caesarean section (CS) is increasing worldwide. While a CS can be life-saving when medically indicated, it can cause adverse health effects for both women and children. This trial aims to evaluate the effect of the smartphone application, which aims to control the gestational weight gain, on the rate of CS in overweight and obese women.

**Methods:**

Overweight and obese primiparas (BMI ≥ 24 kg/m^2^) with age between 20 and 40 years old were recruited at Beijing Obstetrics and Gynecology Hospital, and randomly assigned into the intervention group (143 cases) and the control group (138 cases). The intervention group applied the smartphone application (App) to control gestational weight gain in addition to the usual care, and the control group received the usual care. Primary outcome was cesarean section (CS) rate. Secondary outcomes included gestational hypertension, preeclampsia and eclampsia, gestational diabetes mellitus, postpartum hemorrhage, neonatal asphyxia, and macrosomia.

**Results:**

There was a significant difference in CS rate, with 53.3% in the intervention group and 65.4% in the control group (P = 0.044). The difference still exists in the overweight subgroup (32.6% vs. 55.6%, P = 0.04), but disappears in the obesity subgroup (63.0% vs. 69.1%, P = 0.381). The median of gestational weight gain (GWG) of the intervention group is 8.5 kg (IQR 5.5, 11.0), which is significantly less than that of the control group (median 10.0 kg, IQR [6.0, 14.0], P = 0.008). The intervention group has significantly lower rate of postpartum hemorrhage (5.19%) than the control group (12%) (P = 0.045). There were no significant differences between the groups in gestational hypertension, gestational diabetes mellitus, neonatal asphyxia, and macrosomia.

**Conclusion:**

The smartphone assisted weight control may help reduce CS rate. The effects of the smartphone application might be via the management of gestational weight gain.

**Trail registration:**

This trial was registered at Chinese Clinical Trial Registry. Registration number is ChiCTR2300068845 (retrospectively registered, 01/03/2023).

**Supplementary Information:**

The online version contains supplementary material available at 10.1186/s12884-023-06004-7.

## Introduction

Overweight and obesity in pregnant women is becoming more and more common worldwide [1]. Based on a panel data model, it was estimated that there were more than 4.28 million overweight and obese pregnant women in China in 2014, which increased by 33.9% from 2005, and China was listed as one of the 20 high overweight and obesity burden countries [2]. According to World Health Organization (WHO), overweight is defined as Body Mass Index (BMI) over 25 kg/m^2^, but less than 29.9 kg/m^2^, and obesity is defined as BMI over 30 kg/m^2^. However, for Asian and South Asian populations, this classifications for BMI are slightly different with BMI over 25 kg/m^2^ as obesity [3, 4]. Due to the difference of race and other social-economic factors, in China, overweight is defined as BMI over 24 kg/m^2^, but less than 27.9 kg/m^2^, and obesity is defined as BMI over 28 kg/m^2^ [5].

A number of previous studies showed that overweight and obesity are risk factors of adverse maternal and perinatal outcomes, such as preeclampsia, caesarean section (CS), prematurity and macrosomia [6, 7]. A meta-analysis including 11 cohort studies showed that obesity was an independent risk factor for both elective and emergency CS [8]. In addition to pre-pregnant overweight and obesity, gestational weight gain (GWG) above the recommendation of Institute of Medicine (IOM, 7.0-11.5 kg for overweight and 5.0-9.0 kg for obesity) can also increase the risk of CS. Eloranta AM et al. found that excessive GWG increased the risk of intrapartum caesarean section among normal weight (adjusted odds ratio [aOR] 1.46, 95%CI 1.23–1.73) and overweight women (aOR 1.291, 95%CI 1.04–1.60) [9]. In the Chinese population, the aOR of CS was 1.42 (95% CI 1.06–1.88) for excessive GWG [10]. On the other hand, diet and physical activity based interventions in pregnancy, which can reduce GWG, reduced the odds of CS (0.91, 95% CI 0.83–0.99) in a meta-analysis of 32 studies with 11,410 women [11]. Although a CS can be life-saving when medically indicated, it can cause health effects for both women and children. For women, CS increases the risks of uterine rupture in successive pregnancy, ectopic pregnancy, and preterm birth, while for the children, CS may alter their immune development and intestinal gut microbiota diversity, which lead to long term effects [12, 13]. Overuse of CS, such as CS without medical indication, can also be a waste of human and financial resources. A study covering every county in mainland China’s 31 provinces showed that the CS rate rose from 28.8% in 2008 to 34.9% in 2014 [14]. Thus, interventions are necessary to decrease the risks of CS, especially for overweight and obese women.

Smartphone is getting popular in the past decade and changing the people’s lifestyle. It is now very common to use smartphone to monitor the health status for general people. In addition, smartphone is applied or tested to help manage different types of diseases, such as type 2 diabetes, hypertension and chronic pains [15–17]. There are also some clinical trials to test whether it can assist to control blood glucose level of pregnant women or improve maternal and fetal outcomes [18, 19]. However, such studies were usually executed with pregnant women with gestational diabetes mellitus (GDM) or aimed to investigate the effectiveness on glycemia and/or insulin resistance. The effects of smartphone on CS, especially in overweight and obese pregnant women, is still largely unknown.

In this study, we conducted a randomized controlled trial (RCT) to investigate the effects of a smartphone based weight management application (App) on the CS rate in overweight and obese pregnant women defined by the Chinese criteria.

## Methods

### Study design

We conducted this RCT at Beijing Obstetrics and Gynecology Hospital, Capital Medical University. The participants were recruited from March, 2021 to December, 2021. The participants were randomly assigned to either the intervention group or the control group. Primary outcome is CS rate. Secondary outcomes are gestational hypertension, preeclampsia and eclampsia, GDM, postpartum hemorrhage, neonatal asphyxia, and macrosomia. The trial register number is ChiCTR2300068845 (01/03/2023).

### Definition of the outcomes

1. Gestational hypertension, preeclampsia and eclampsia: gestational hypertension presents after 20 gestational weeks (blood pressure [BP] ≥ 140/90 mmHg), recovers within 12 weeks after delivery, and is with negative proteinuria. Preeclampsia is gestational hypertension associated with significant proteinuria. Eclampsia occurs when convulsions are associated with preeclampsia.
2. GDM: diabetes occurs during pregnancy. It is diagnosed using a 75 g oral glucose tolerance test (OGTT). GDM can be diagnosed if the fasting glucose is > 5.1 mmol/L, or the level 1 h after the glucose challenge is > 10.0 mmol/L or the level 2 h is > 8.5 mmol/L.
3. Postpartum hemorrhage: blood loss of more than 500mL following vaginal delivery or more than 1000mL following CS within 24 h.
4. Neonatal asphyxia: Apgar 1 min ≤ 7.
5. Macrosomia: fetal weight is over 4000 g.

### Study participants

Inclusion criteria: (1) pre-pregnant BMI ≥ 24 kg/m^2^; (2) age between 20 and 40 years old to meet the major population of pregnant women in our hospital who undertake CS following the standard indications; (3) no previous history of childbirth; (4) singleton pregnancy; (5) routine antenatal care from 12 gestational weeks until delivery in our hospital; (6) full term delivery.

Exclusion criteria: (1) pre-pregnant diabetes, hypertension and/or other cardiovascular diseases; (2) complications with systemic lupus erythematosus and other autoimmune diseases; (3) multiple pregnancy; (4) preterm delivery.

### Randomization and blinding

The participants were randomly assigned either to the intervention group (App + usual care) or the control group (usual care only) in a 1:1 ratio. A blocked randomization program (block size of 12) was created with a computer-generated list of random numbers. The participants and obstetricians at the hospital were not blinded.

### Sample size calculation

The sample size was calculated based on CS rate, which is the primary outcome of this trial. According to previous studies, the difference of CS rate between proper and improper gestational weigh gain of overweight or obese pregnant women is 15–20%. 15% was taken for a two-sided test (α = 0.05 and β = 0.01). The sample size was calculated with the formula n = 2pq(Zα + Zβ)^2^/(P1-P2)^2^. 260 participants (130 participants per group) are necessary.

### The intervention

**Fig. 1:**
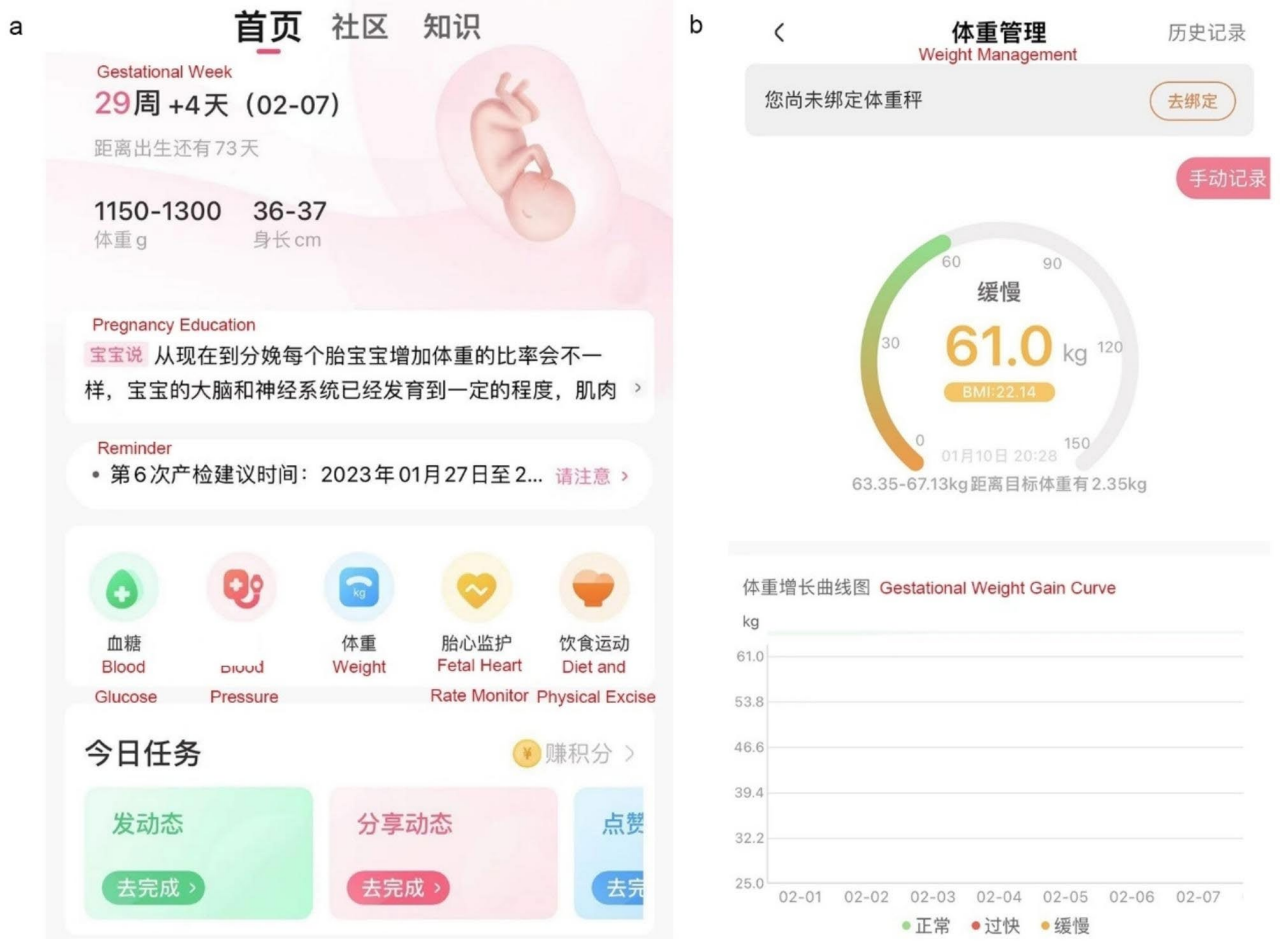
The home menu of the App **(a)** and page of weight management **(b)**. The names of key features are translated into English, and shown in red

The pregnant women in the intervention group took the smartphone assisted weight management in addition to the usual care. The participants downloaded the App at the hospital or home. The pregnant women were asked to use the App from the establishment of their gestational records at the hospital (6–7 weeks). The App can run in either iOS or Android system. The App has the following main icons (Fig. 1):

1. Weight management plan: according to the user’s BMI and gestational week, the system automatically and personally recommends weight gain plan, and indexes of calorie intake and consumption. The indexes are specified in each gestational week and serve as the references of the objectives of daily weight gain, diet and physical exercise.
2. Daily recording: (a) weight recording: the user enters the daily weight, and gets automatic feedback according to the gestational week; (b) diet recording: the user enters the grams and categories of food for each meal. The system automatically calculates the calorie intake; (c) physical excise recording: the user authorizes the App to obtain the pedometer data from the operation system and the App automatically converts them to calorie consumption. The user enters the types of physical activity (PA) and time, and the App calculates the calorie consumption, respectively.
3. Data trend: the App generates the curves of weight, diet and exercise, and inspires the user to follow the weight management plan.
4. Reminder: (a) antenatal care reminder: according to the user’s gestational week, the App reminds the user to take standard antenatal care at the hospital; (b) recording reminder: the App reminds the user to enter daily weight, diet and exercise data.
5. Pregnancy education: according to the user’s gestational week, the App delivers the pregnancy education contents.

The participants were asked to report their weight, diet and PA via the App at least once a week. We define the termination of the App usage as the gestational weeks in which the patients entered their weight, diet and/or physical activity data for the last time. The period in which the patients were supposed to use the App is the time between the record establishment at hospital and termination of the App usage. We define the adherence as below,

$${\rm{Adherence}} = \frac{{{\rm{weeks\, in\, which\, the\, user\, reports\, via\, the\, App\, at\, least\, once}}}}{{{\rm{weeks\, between\, delivery\, record\, establishment\, at\, hospital\, and\, delivery}}}}$$

The frequent user is defined as adherence ≧ median of adherence, and the non-frequent user is defined as adherence < median of adherence.

### Usual care

The weight management in the first trimester includes: (1) learning diet pagoda; (2) recommendations on GWG and exercise; (3) management of exogenous dietary supplements.

The weight management in the second trimester includes: (1) instructing pregnant women to evaluate GWG; (2) guiding the pregnant women to discover the questions and communicate with obstetricians; (3) explaining examine time and method of GDM.

The weight management in the third trimester includes: (1) instructing the pregnant women to evaluate the nutrition status; (2) instructing pregnant women to evaluate the GWG; (3) guiding the pregnant women to discover the questions and communicate with obstetricians.

### Statistic analysis

Continuous data were analyzed using the t-test when the data were normally distributed or the Mann-Whitney-Wilcoxon test when the distributions were skewed. Categorical data were compared using χ^2^ test. Analyses were performed using SPSS statistics V.22.0.

## Results

**Fig. 2:**
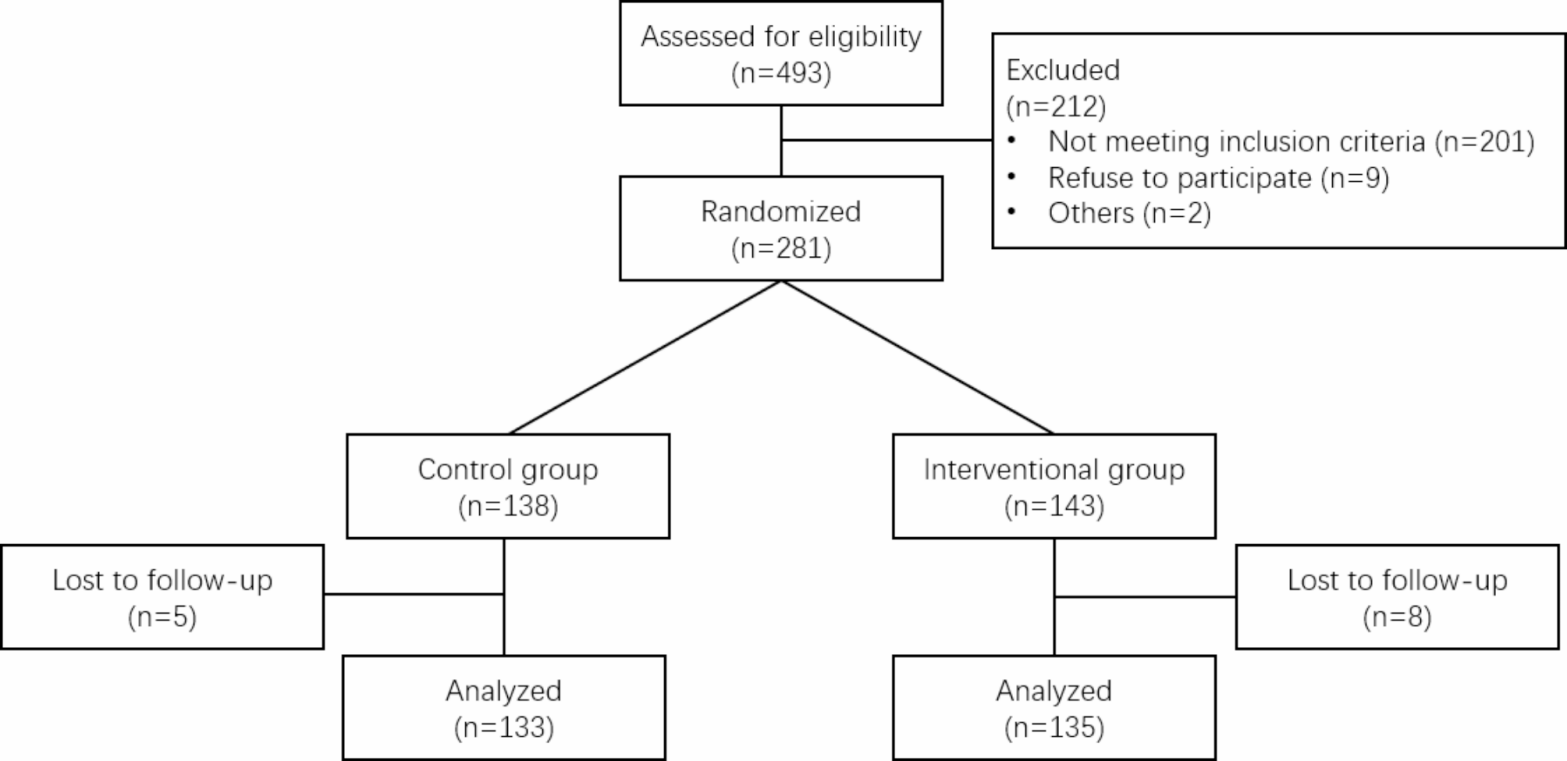
the flow chat of the trial

From March, 2021 to December, 2021, 281 overweight and obese pregnant women were enrolled, among whom 143 participants were assigned to the intervention group and 138 participants were assigned to the control group. 8 and 5 women were lost to follow up respectively in the intervention and control group. The data from 135 women in the intervention group and 133 women in the control group were finally analyzed. The detailed flow chat is shown in Fig. 2. At the baseline, there were no significant differences of age, gestational weeks and pre-pregnancy BMI between the groups, and the proportions of overweight and obese women in each group are comparable, as shown in Table 1. The median of termination of the App usage is 35 (interquartile rage [IQR] 29, 38). The median of period in which the patients were supposed to use the App is 29 weeks (IQR 23, 32). During the trial, the median of GWG of the intervention group is 8.5 kg (IQR 5.5, 11.0), which is significantly less than that of the control group (median 10.0 kg, IQR [6.0, 14.0], P = 0.008). When the participants were stratified, in the overweight subgroup, the median of GWG of the intervention group is significantly less than that of the control group, while there is no difference of medians of GWG between the groups in the obesity subgroup (Table 2). Meanwhile, significant higher percentages of overweight women are with inadequate or recommended GWG in the intervention group, compared to the control group. However, although the percentage of obese women with excessive GWG of the intervention group is lower than that of control group, the difference is not significant (Supplementary Table 1).

**Table 1:** Baseline data of the control group and intervention group

	the control group (n = 133)	the intervention group (n = 135)	P value
age (y)	33.0 (31.0, 36.0)	32.0 (30.0, 35.0)	0.211
pre-pregnant BMI (kg/m^2^)	29.34 (27.84, 31.23)	28.9 (27.63, 30.86)	0.486
gestational week (w)	39 (38, 39)	39 (38, 40)	0.271
overweight number (%)	36 (27.1)	43 (31.9)	0.391
obesity number (%)	97 (72.9)	92 (68.1)	

Median (IQR)

**Table 2:** Gestational weight gain of the control group and the intervention group

	the control group	the intervention group	P value
Overall median of Gestational Weight Gain (kg, IQR)	10 (6, 14)	8.5 (5.5, 11)	0.008
stratification			
Median of GWG in overweight (kg, IQR)	11.25 (5.25, 16.5)	8 (5.4, 10.2)	0.035
Median of GWG in obesity (kg, IQR)	9.7 (6, 13)	8.5 (6, 11)	0.102

For the CS rate, the application of smartphone assisted weight control can significantly reduce it from 65.4% in the control group to 53.3% in the intervention group (P = 0.044, Table 3). The indications for CS are summarized in supplementary Table 2. This difference of the CS rate still exists in the overweight subgroup, but disappears in the obesity subgroup (Table 4). 61 (45.86%) women in the control group and 63 (46.67%) women in the intervention group undertook induced labor, which had no difference (P = 0.895). For the rate of CS in labor and CS after failure of induced labor, there are no significant differences between the two groups (Table 3).

**Table 3:** Primary and secondary outcomes of the control group and intervention group

	the control group (n = 133)	the intervention group (n = 135)	P value
cesarean section (%)	87 (65.4)	72 (53.3)	0.044
cesarean section in labor (%)	18 (13.4)	17 (12.6)	0.684
cesarean section after failure of induced labor (%)	18 (13.4)	13 (9.6)	0.318
gestational hypertension (%)	30 (22.6)	32 (23.7)	0.824
gestational diabetes mellitus (%)	53 (39.8)	52 (38.5)	0.823
postpartum hemorrhage (%)	16 (12.0)	7 (5.19)	0.045
neonatal asphyxia (%)	3 (2.3)	1 (0.7)	0.303
macrosomia (%)	13 (9.8)	17 (12.6)	0.464

**Table 4:** Primary outcome in overweight and obesity subgroup

stratification	group	cesarean section (%)	P value
overweight	control (n = 36)	20 (55.6)	0.04
	intervention (n = 43)	14 (32.6)	
obesity	control (n = 97)	67 (69.1)	0.381
	intervention (n = 92)	58 (63.0)	

The median of adherence to the App is 0.63 (IQR 0.37, 0.78), which is used as a threshold to divide the intervention group into frequent user subgroup (≥ 0.63) and non-frequent user subgroup (< 0.63). There are 66 non-frequent users and 69 frequent users. The median of the GWG of frequent users is 7.5 kg (IQR 5, 10), which is significantly lower than that of non-frequent users is 9.5 kg (IQR 7,11.62, P = 0.014). The CS rate of the frequent user group showed a trend to be lower than that of non-frequent user group (46.38% vs. 60.61%, P = 0.098, Table 5), but is significantly lower than that of the control group (46.38% vs. 65.41%, P = 0.009). On the other hand, the CS rate of non-frequent user is comparable to that of the control group (60.61% vs. 65.41%, P = 0.506).

**Table 5:** CS in different frequent user subgroups

	CS		
	Yes	No	P value
Frequent User	32	37	0.098
non-Frequent User	40	26	

For the secondary outcomes, the App intervention can significantly reduce the rate of postpartum hemorrhage from 12.0% in the control group to 5.19% in the intervention group (P = 0.045). However, the differences of gestational hypertension, pre-eclampsia and eclampsia, GDM, neonatal asphyxia, and macrosomia are not significant between the two groups (Table 3). The birth weight has a trend to be lower in the intervention group (median 3390 g IQR [3105, 3670]) than the control group (median 3470 g (3250, 3740), P = 0.056).

## Discussion

In this study, our data showed that smartphone assisted weight management can help reduce the CS rate in overweight pregnant women, but not obese ones. The rate of postpartum hemorrhage is also lower in the intervention group, compared to the control group. However, the differences of gestational hypertension, GDM, neonatal asphyxia, and macrosomia are not significant between the two groups.

Maternal obesity is an independent risk factor of adverse pregnancy outcomes, such as pre-eclampsia, CS and macrosomia. Both in Western countries and China, overweight and obese women were more likely to require a CS, compared with those of normal weight, and the risk of obesity is even higher than overweight [20, 21]. Smartphones are widely used nowadays and capable to change the people lifestyle, by monitoring exercise frequency, nutrition intake, weight gain, *etc*. A previous study showed that App monitor can lower the rate of emergency CS in pregnant women with GDM, but this effect disappeared when the patients were stratified by parity [18]. However, the CS rates were not significantly different between the intervention group and the control group in other RCTs with patients with GDM [19, 22]. A recent meta-analysis, pooling eight studies and including 1,409 participants, also reported that insignificant differences between the control and intervention groups were found in risks of cesarean birth [23]. However, all these studies were designed with the primary outcome to control blood glucose level, and their participants were all with GDM and covered all BMI categories from underweight to obesity. Thus, our study is the first one to explore the effects of App monitor on the CS rate in overweight and obese pregnant women.

How smartphone assisted weight control can affect CS rate remains largely unclear. However, our data suggest that the App intervention might function via GWG restriction. Several studies showed excessive GWG resulted in higher CS rate. A meta-analysis, including 23 studies and over 1.3 million women, reported that GWG above Institute of Medicine (IOM) guidelines was associated with higher risk of CS for overweight women (OR, 1.29 [95% CI, 1.21–1.39]) and obese women (OR, 1.22 [95% CI, 1.05–1.42]) [24]. In our study, in the overweight subgroup, the median of GWG of the interventional group is significantly less than that of the control group, even though both of them were within the recommendations of IOM (7.0-11.5 kg). Meanwhile, significant higher percentages of overweight women were with inadequate or recommended GWG in the intervention group, compared to the control group. This may help explain the significant difference of CS rate between the two groups in the overweight women. On the other hand, in the obesity subgroup, although the median of GWG of the intervention group was within the recommendation of IOM (5.0-9.0 kg), and that of the control group was above the recommendation, the difference is not significant statistically. Meanwhile, the percentages of obese women within different GWG categories were similar between the control and interventional group. This may explain why we found no difference of CS rate in the obese subgroup. In addition, it is well accepted that excessive GWG may increase odds of CS, but this effect may differ across all different pre-pregnant BMI categories. For example, Rogozinska E. et al. showed that aORs of CS are 1.68 (95%CI 1.19–2.35) and 1.44 (95%CI 1.10–1.89) for overweight and obese women [25]. Another meta-analysis by Su L. et al. also showed the pooled ORs of CS are 1.27 (95%CI 1.20–1.33) for obesity class I, 1.21 (95%CI 1.005–1.46) for class II and 1.10 (95%CI 1.001–1.22) for class III [26].

In this study, the adherence we used is only a rough estimation of the frequency the participants used the App. Because we do not design a questionnaire to learn directly from them about the intensity they use the App and how strictly they follow the advice provided by the App, we do not exactly know how strong the App affects their lifestyle to control GWG. Nevertheless, the women who used the App more frequently had less GWG. This is consistent with other studies which try to explore the effects of digital health interventions on weight control [27, 28]. Meanwhile, we observed that CS rate of the frequent users has a trend to be lower than that of the non-frequent users, which is comparable to CS rate of the control group. Although we cannot decide whether a minimum adherence of 0.63 may guarantee the effect of the App intervention on CS rate, this result suggests that the App might help reduce the CS rate in overweight and obese women. It also indicates that a high adherence is necessary for such lifestyle intervention trials.

In addition to CS rate, we also found lower rate of postpartum hemorrhage in the intervention group than the control group. According to a recent systematic review and meta-analysis, six variables were found to be definite risk factors for postpartum hemorrhage, which were Asian race, a history of prior postpartum hemorrhage, preexisting or gestational diabetes mellitus, placental disorders and prolonged labor. Nine additional variables were likely its associated factors, such as Hispanic ethnicity, nulliparity, hypertensive diseases of pregnancy, multiple gestation, chorioamnionitis, uterine rupture, predelivery oxytocin exposure, induction of labor, and instrumented vaginal delivery [29]. However, CS is also included in some risk prediction models of postpartum hemorrhage [30, 31]. As some of the risk factors analyzed are interactive with each other, such as prolonged labor with CS, and GWG and obesity with GDM, we are not sure whether the reduction of the rate of postpartum hemorrhage may result from lower CS rate or less GWG. Future specific designed case-control studies in larger cohort are necessary to further figure out the risk factors of postpartum hemorrhage. .

Although GWG is associated with GDM and excessive GWG increase the risk of GDM [32, 33], lifestyle interventions, such as PA and/or diet control, do not always affect GWG and GDM simultaneously. A meta-analysis including 23 studies with a total of 8877 overweight and obese participants indicated that PA and diet plus PA intervention only had a trend to be the protective factors of GDM, even though they were significantly beneficial for GWG control [34]. Overweight and obese women deposit more adipose tissue in the body, which affects the production of adipokines, cytokines and chemokines, exaggerating the insulin resistance during pregnancy and leading to increased risk to develop GDM [35]. The duration of lifestyle intervention in pregnancy is relatively short, and may not be enough to change the metabolic background of these women [36]. Thus, in addition to the intervention during pregnancy, the clinicians and the community should stress more on the weight control before conception. A population-based cohort study with more than 226 thousand participants showed that a 10% difference in pre-pregnancy BMI was associated with at least 10% lower risk of GDM [37].There are also some limitations for this study. First, all the participants were recruited in a single center in Beijing, a major developed city in China. Thus, the results may not be generalized to other regions, especially the rural regions. Second, some social-economic factors, which may affect the lifestyle, are not collected or not accurate enough to report, such as education, income, and job. Third, although the App provides the function to directly communicate with the obstetricians, considering the doctor’s workload, most of the feedback to the users’ questions and antenatal education were completed automatically by the App. This may affect the compliance of the participants. The future study to explore the compliance of this smartphone assisted weight control is necessary.

## Conclusions

Our study showed that the smartphone assisted weight control may help reduce CS rate. As the effects of this intervention might result from less GWG, it will be interesting to know whether it can help improve other adverse maternal and neonatal outcomes in a larger population in future.

### Electronic supplementary material

Below is the link to the electronic supplementary material.

Supplementary Material 1

## Data Availability

Data can be obtained from the corresponding author upon reasonable request.
