# Supplementary material for: Effect of the smartphone application on caesarean section in women with overweight and obesity: a randomized controlled trial in China
Source: BMC Pregnancy Childbirth. 2023 Oct 23;23:746. doi: 10.1186/s12884-023-06004-7 (PMC10594860; doi:10.1186/s12884-023-06004-7)
Supplement: Supplementary file 1 — Supplementary Material 1 [file 12884_2023_6004_MOESM1_ESM.docx]

Supplementary Table 1. The percentages of different GWG categories in the control and intervention group

|  | the control group | the interventional group | P value |
| --- | --- | --- | --- |
| overweight (n, %)* |  |  |  |
| inadequate GWG | 10 (27.78) | 14 (32.56) | 0.017 |
| recommended GWG | 10 (27.78) | 22 (51.16) |  |
| excessive GWG | 16 (44.44) | 7 (16.28) |  |
| obesity (n, %)** |  |  |  |
| inadequate GWG | 16 (16.49) | 20 (21.74) | 0.487 |
| recommended GWG | 31 (31.96) | 32 (34.78) |  |
| excessive GWG | 50 (51.55) | 40 (43.48) |  |
| * in overweight subgroup, inadequate GWG <7.0kg, recommended GWG 7-11.5kg, excessive GWG >11.5kg | | | |
| ** in obesity subgroup, inadequate GWG <5.0kg, recommended GWG 5-9kg, excessive GWG >9.0kg | | | |

Supplementary Table 2. Indications of CS

| indication of the CS | the control group | the interventional group |
| --- | --- | --- |
| fetal distress | 25 | 25 |
| failed induction | 18 | 13 |
| cephalopelvic disproportion | 11 | 7 |
| breech | 8 | 8 |
| previous uterine or cervix surgery | 5 | 4 |
| gestational hypertension and pre-eclampsia | 5 | 3 |
| placenta previa | 4 | 1 |
| placental abruption | 0 | 1 |
| transvers lie | 0 | 1 |
| others* | 11 | 9 |
| other indications include: oligohydramnios, biliary colic, macrosomia, advanced maternal age, lumbar herniated discs, fetal growth restriction | | |
